# Investigation Into the Dynamics of the Cupula in the Vestibular Organ of Adult Zebrafish Using Metabolic Glycoengineering

**DOI:** 10.1002/anie.202515593

**Published:** 2026-01-20

**Authors:** Hans Scherer, Andrea Jüngst, Verena F. Schöwe, Anne‐Katrin Gronewald, Valentin Wittmann

**Affiliations:** ^1^ Department of Otolaryngology Head and Neck Surgery and Institute of Clinical Chemistry and Pathobiochemistry Charité‐Universitätsmedizin Berlin 13353 Berlin Germany; ^2^ Department of Chemistry and Konstanz Research School Chemical Biology (KoRS‐CB) University of Konstanz Universitätsstraße. 10 78464 Konstanz Germany

**Keywords:** Bioorthogonal chemistry, Inner ear, Metabolic glycoengineering, Sudden loss of vestibular function, Vestibular neuropathy

## Abstract

Sudden loss of peripheral vestibular function is a common clinical disorder. The primary cause of this disorder is not known. Previous experiments in pigeons showed that an induced mechanical leakage in the cupula of a semicircular canal causes symptoms equivalent to those observed in humans after sudden loss of peripheral vestibular function. The cupula is an acellular membrane, which is critical for the detection of angular acceleration of the head. It consists of the matrix glycoprotein cupulin, which is secreted by supporting cells in the crista ampullaris. Currently, it is unclear whether cupulin is continuously produced resulting in permanent cupular neogenesis. Such a process could explain recovery of function observed in many patients. We applied metabolic glycoengineering to demonstrate the existence of cupulin renewal. Intraperitoneal injection of *N*‐azidoacetylgalactosamine (GalNAz) into zebrafish leads to incorporation of this sugar in the cupula. Preparation of the cupula after various time intervals followed by fluorescence labeling by click chemistry, resulted in a distinct band within the cupula visible in fluorescence microscopy. Time‐delayed double injection gave rise to two bands. A long‐term experiment allowed to estimate that complete renewal of the zebrafish cupula occurs over a cycle of eight to ten weeks.

## Graphical abstract

The cupula is a membrane within the vestibular organ that senses rotatory accelerations of the head. Metabolic glycoengineering in combination with bioorthogonal labeling demonstrates that in adult zebrafish – being a model for the human inner ear – this membrane is constantly renewed. This provides an explanation for recovery of patients suffering from sudden loss of peripheral vestibular function.

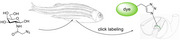

## Introduction

Sudden loss of peripheral vestibular function resulting in severe vertigo, postural instability, sudden loss of hearing, or combinations thereof is a common clinical disorder.^[^ ^1^ ^]^ The loss of function can be partial and in some cases complete. To date, the clinical outcome cannot be predicted. In some cases, spontaneous partial or total recovery of the function is observed; in other cases no improvement occurs.^[^ ^2^ ^]^ Clinical experience indicates that those patients with total loss of labyrinth and cochlea function rarely recover. Due to the limited knowledge of the pathophysiology of such disorders, the therapeutic regimes remain largely ineffective. Current, albeit scarcely substantiated, explanations, for this sudden, traumatic damage are disturbances of the blood supply^[^ ^3^ ^]^ or an infection of the ganglion of the vestibular nerve with herpes simplex virus type 1.^[^ ^3^, ^4^ ^]^ Surprisingly little is discussed, or known, about mechanical problems in the extremely complex structure of the inner ear. Of the various structures, two parts are particularly likely to trigger a sudden loss of function, namely the cupula in the vestibular organ and the tectorial membrane in the cochlea. The investigation presented here is directed at the cupula, a membranous structure in the semicircular canals of the vestibular organ. These sub‐organs perceive rotatory accelerations of the head about the three main axes, thus contributing to the maintenance of balance and spatial orientation.

**Figure 1:**
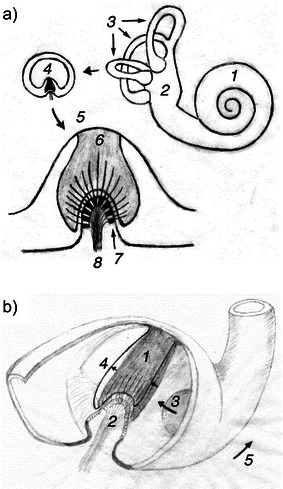
Simplified schematic drawing of the human inner ear. a) Illustrated are the cochlea (*1*), the vestibule (*2*), and the layout of the three near‐orthogonal semicircular canals (*3*). The enlargement indicates the semicircular duct (*4*) which is filled with endolymph fluid and occluded by the cupula, an acellular membrane located in the widened section, called ampulla. The enlargement of the ampulla (*5*) shows the cupula (*6*), hair cells (*7*), and vestibular nerve fibers (*8*). b) Cross‐section of the ampulla. The cupula (*1*, dark area) rides on the crista ampullaris (*2*) which hosts the population of sensory hair cells and supporting cells. Hairs protruding from the sensory cells partly extend into the cupula. A rotatory acceleration of the head generates a shift of the endolymph (*3*) in the plane of the canal. This fluid shift distends the cupula toward the side (*4* and short arrows) opposite to the direction of the acceleration (*5*). The sensory hairs are thus deflected and open ion canals in the sensory cells. Potassium ions from the endolymph enter the hair cells, leading to a generator potential of the cell and in consequence to a change in the firing rate of action potentials in the afferent vestibular nerve.

The cupula is a membrane in a widened section of a semicircular canal, named the ampulla (Figure 1).^[^ ^5^ ^]^ It is sensitive to fluid shift during angular acceleration of the head. The shift of fluid displaces the membrane, and its movement is transduced by a population of hair cells located at its base.^[^ ^6^, ^7^, ^8^ ^]^ The watertight adherence of the cupula to the ampullar wall is essential for the proper function of the semicircular canal. Any leakage of the membrane would cause some loss of function. In a chronic animal (pigeon) model, an induced leakage through mechanical detachment of the cupula from the ampullar wall caused symptoms equivalent to those observed in humans after a sudden loss of peripheral vestibular function.^[^ ^9^, ^10^ ^]^ These experiments suggest that the sudden loss of vestibular function observed in humans might be due to any leakage caused by spontaneous detachment of the cupula from the ampullar wall. Several possibilities for spontaneous detachment are discussed, including hyperpressure (hydrops) in the inner ear,^[^ ^11^, ^12^ ^]^ which might lift the ampullar roof. Other possibilities are structural problems of the cupula itself.

The cupula is an acellular structure that consists mainly of the matrix protein cupulin which is secreted as a pro‐protein by supporting cells in the crista ampullaris, the sensory cell area.^[^ ^13^ ^]^ The pro‐protein is converted to the active matrix protein by a protease. The existence of multiple, presumably secretory vesicles in the supporting cells supports the hypothesis that there is a continuous process of cupular neogenesis. Accordingly, any interruption of this process would result in structural deficits in the cupula. Such a continuous process could be disturbed either at the level of the biochemical production of cupulin, or at the level of the protease which converts the secreted pro‐protein to the active protein. A continuous process of cupular neogenesis could also be a possible explanation for the recovery of the function observed in some cases. However, currently we do not have any knowledge on cupulin renewal.

To determine the existence of such a hypothetical cupulin renewal, a method is required that enables the observation of cupular growth over time. A potential approach is to perform a pulse‐chase experiment that allows labeling of cupulin as the major component of the cupula at a certain point of time followed by observation of cupulin regeneration. To this end, a method to metabolically label cupulin with a fluorescent marker at a defined point of time was chosen. Preparation of the vestibular organ including the cupula after varying time intervals followed by fluorescence microscopy analysis was performed to determine whether any growth occurred and, if so, over which time scale. We decided to investigate the cupula in adult zebrafish. The zebrafish is an ideal model organism to study cupulin renewal because the anatomy of its vestibular organ in the area of the cupula is very similar to that of humans.^[^ ^14^ ^]^ Since it is not embedded in bone, it is easily accessible, and the cupula is clearly visible in cross sections of the head like it has also been shown for preparations of the vestibular organ of salmon.^[^ ^13^ ^]^

Earlier studies have shown that PNGase F treatment of cupulin lowers its molecular weight, indicating that cupulin is a glycoprotein containing *N*‐linked glycans.^[^ ^13^ ^]^ This observation provides the opportunity to label cupulin by metabolic glycoengineering (MGE). MGE is a method for the visualization of glycans both in cell culture and in living organisms.^[^ ^15^, ^16^, ^17^, ^18^ ^]^ In this approach, chemically modified monosaccharides bearing a chemical reporter group are applied to cells or organisms, metabolized by the biosynthetic machinery, and incorporated into glycoconjugates including glycoproteins. The reporter group can subsequently react in a bioorthogonal ligation reaction^[^ ^19^, ^20^ ^]^ allowing the introduction of a functional probe, such as an enrichment tag for proteomics analyses or a fluorescence label for visualization of glycans. Previously, the method was used to monitor the glycome in different settings from cell culture, including intracellular labeling of glycoproteins,^[^ ^21^ ^]^ to living animals including mice.^[^ ^22^ ^]^ In developing zebrafish embryos, azide‐containing derivatives of GalNAc,^[^ ^23^, ^24^ ^]^ fucose,^[^ ^25^ ^]^ ManNAc,^[^ ^26^, ^27^ ^]^ and sialic acid^[^ ^26^ ^]^ have been employed. MGE leads to labeling of the whole glycome. Although protein‐specific labeling of glycans has been reported before,^[^ ^28^, ^29^, ^30^, ^31^, ^32^ ^]^ these techniques are dispensable for visualizing cupula growth since it consists mainly of cupulin.

Here we report the application of MGE to label the cupula in adult zebrafish. We found that several days after injection of a single dose of a suitable carbohydrate derivative a distinct band in the cupula became visible in cross sections of the zebrafish head. The position of the band progresses over time between the carbohydrate injection and the preparation of the cupula, thus permitting direct visualization of the growth of the cupula. These observations demonstrate for the first time that cupulin renewal is a continuous process. A long‐term observation over several weeks indicates that the complete renewal of the zebrafish cupula occurs within approximately eight to ten weeks.

## Results and Discussion

### GalNAz Is Suitable for Labeling of the Cupula

**Figure 2:**
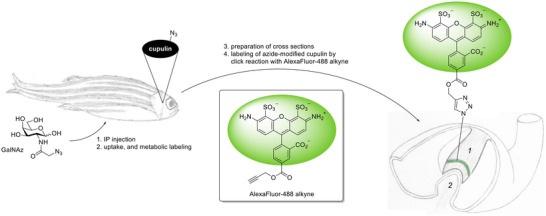
Metabolic glycoengineering with adult zebrafish. A sugar derivative (GalNAz) was intraperitoneally injected, taken up by the supporting cells, metabolized, and incorporated into glycoproteins, among them cupulin. After sample preparation, the azide‐modified glycoproteins were ligated by CuAAC to AlexaFluor‐488 alkyne allowing visualization of labeled cupulin by fluorescence microscopy. During the preparation of cross sections, the cupula shrinks and changes its appearance (cf. Figure 3 and previous work^[^ ^13^ ^]^) *1* = cupula, *2* = crista ampullaris.

To monitor growth of the cupula we applied MGE to adult zebrafish. We performed the experiments in adult animals because the disease of sudden loss of function in humans mainly occurs in adults. In addition, we wanted to avoid interference with adolescent structures. Our experiments should clarify whether the cupula can be regarded as a permanent structure or if there is a continuous, lifelong renewal of cupulin. Various chemical methods have been described for bioorthogonal labeling of glycans, for example copper(I)‐catalyzed azide‐alkyne cycloaddition (CuAAC)^[^ ^33^, ^34^ ^]^ or tetrazine ligation.^[^ ^32^, ^35^, ^36^, ^37^ ^]^ Here we used CuAAC (also known as click reaction)^[^ ^38^, ^39^ ^]^ in which an azide reacts with a terminal alkyne forming a triazole. To apply the azide‐labeled sugar derivative, a small volume (a few µL) of a solution of the azide‐labeled sugar derivative was intraperitoneally injected into anaesthetized fish. Although the very first MGE experiments had been carried out with unprotected ManNAc derivatives,^[^ ^40^ ^]^ it became common to use peracetylated carbohydrate derivatives for MGE because these nonpolar derivatives are cell membrane permeable increasing cellular uptake.^[^ ^41^ ^]^ Unprotected carbohydrate derivatives have to be applied in much higher concentrations to achieve cellular uptake.^[^ ^42^ ^]^ Their effectiveness for metabolic labeling is dependent on the type of derivative and cell type.^[^ ^43^ ^]^ Peracetylated carbohydrate derivatives, however, can result in nonspecific attachment to cysteine side chains of proteins in a non‐enzymatic reaction (*S*‐glyco modification)^[^ ^44^ ^]^ implicated in neurotoxicity.^[^ ^45^ ^]^ Furthermore, the low solubility of these derivatives require large injection volumes not beneficial for the fish. Accordingly, we decided to employ unprotected sugars for our experiments, which have a high water solubility and do not lead to *S*‐glyco modification.^[^ ^46^ ^]^ The azide‐labeled sugars were used as a 250 mM solution in phosphate buffered saline (PBS) and typically 5 µL of this solution were injected in anaesthetized fish corresponding to 1.25 µmol sugar per gram fish. After recovery, the fish were replaced in the aquarium tank for a defined number of days before being sacrificed. Each fish head was embedded in paraffin and cross sections in the sagittal plane were made. After deparaffinization with glycol and ethanol, the native slices were stained by reaction with AlexaFluor‐488 alkyne and kept in darkness for fluorescence microscopy (Figure 2).

We tested three azide‐labeled sugar derivatives for MGE (*N*‐azidoacetylmannosamine, *N*‐azidoacetylglucosamine (GlcNAz), and *N*‐azidoacetylgalactosamine (GalNAz)) and found that only GalNAz gave an intense, distinct labeling of the cupula. Previously, it was reported that GalNAz is metabolically incorporated into mucin‐type O‐glycans.^[^ ^47^ ^]^ Thus, a possible explanation of our results could be that cupulin, beside the reported N‐glycosylation, is also O‐glycosylated. On the other hand, it is also possible that biochemically formed UDP‐GalNAz is epimerized to UDP‐GlcNAz and then incorporated in other types of glycans^[^ ^48^ ^]^ or that GalNAz labels a minor protein component of the cupula that is O‐glycosylated.

**Figure 3:**
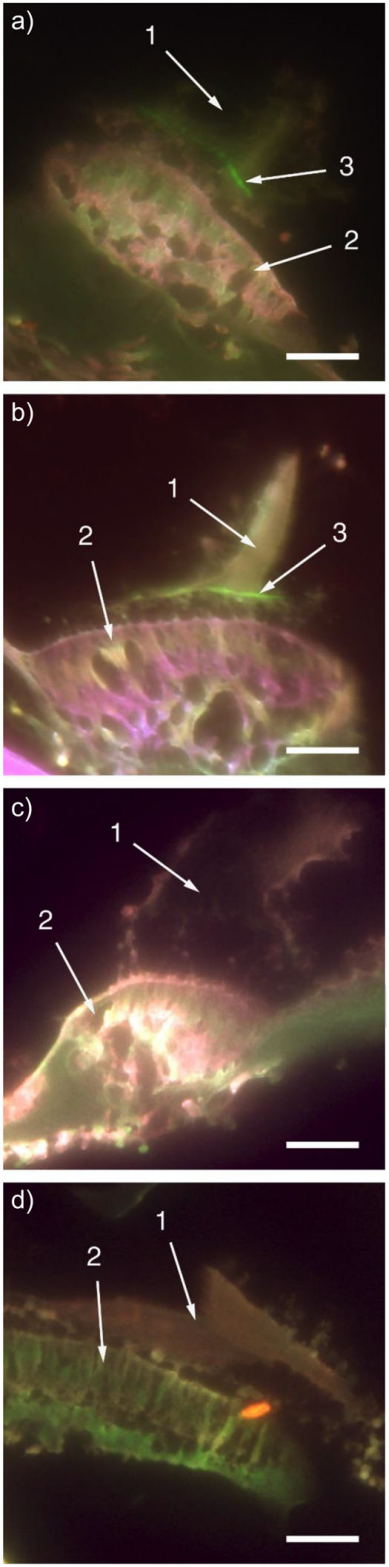
Cupula preparations of zebrafish visualized by fluorescence microscopy. In each picture “1” marks the cupula (shrunk during the process of histology) and “2” the crista ampullaris with the sensory cells and the supporting cells. a) A fish was intraperitoneally injected with GalNAz. After 4 days, the cupula was prepared and incorporated GalNAz was visualized by click reaction with AlexaFluor‐488 alkyne. GalNAz is visible as a green band marked “3”. b) Another specimen of a zebrafish was sacrificed 4 days after injection of GalNAz and labeled with AlexaFluor‐488 alkyne. The green band of labeled GalNAz (marked “3”) is clearly visible in the cupular material. c) Control 1: Cupula of a zebrafish prepared 4 days after injection of GalNAz. The click reaction was not performed on the cross section. A green band is not visible. d) Control 2: Cupula of a zebrafish which was not injected with GalNAz but the cross section was incubated with AlexaFluor‐488 alkyne. A green band is not visible. Scale bars: 0.1 mm.

With GalNAz and a delay of four days between injection and preparation of the cupula we detected a narrow green band in the cupula slightly above the crista ampullaris (Figure 3A). This finding indicates that GalNAz is resorbed from the peritoneum and is indeed incorporated into the cupula. The experiment was successfully repeated several times (Figure 3B). To ensure that the green band is not an artifact, e.g. due to auto‐fluorescence of the fish tissue, fish were injected with GalNAz but not reacted in the click reaction. In this case, no green band could be detected (Figure 3C). As a second control experiment, zebrafish were injected with phosphate buffered saline not containing GalNAz but cross sections of the head were incubated with AlexaFluor‐488 alkyne. These samples did not show a comparable green staining either (Figure 3D). Thus, we concluded that the green band originates from incorporated GalNAz which was labeled by the click reaction with AlexaFluor‐488 alkyne. To our surprise, the green band was very distinct and sharp, indicating that the unnatural GalNAz was incorporated in significant amounts into the cupula for only a short period of time. Glycoproteins synthesized before and after the period in which GalNAz was present did not contain the azide moiety and were, consequently, not visualized in the labeling process. This appearance of the narrow band is intensified by the fact that the cupula shrinks during the preparation process and thus condenses stained areas of the membrane.

### Time‐Delayed Double Injection of GalNAz Results in Two Stained Bands Revealing Growth Speed

**Figure 4:**
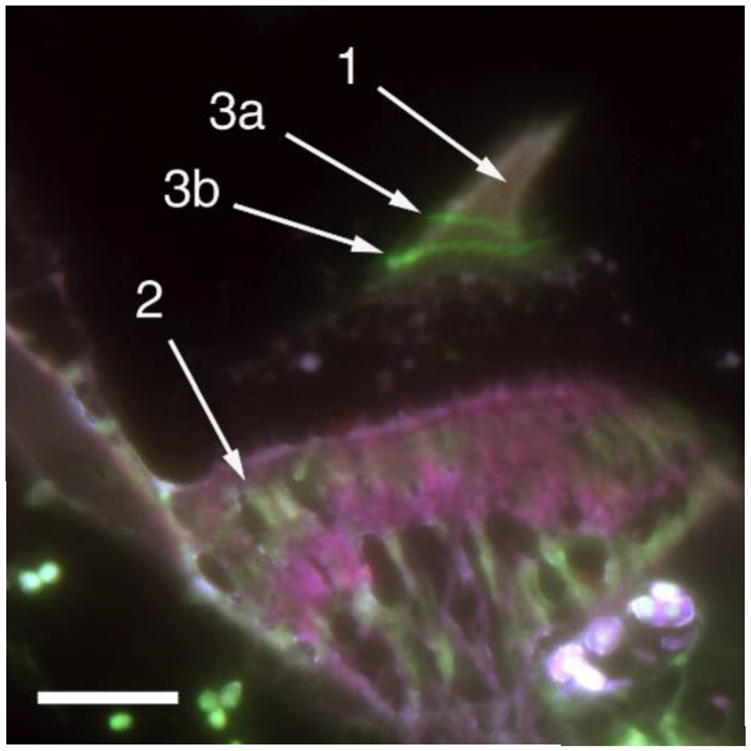
Cupula of a zebrafish after two intraperitoneal GalNAz injections separated by an interval of 13 days. 31 days after the second injection the prepared cross sections were stained with AlexaFluor‐488 alkyne. Two bands are visible, each resulting from one injection of GalNAz. 1 = cupula, 2 = crista ampullaris, 3 = green bands from first (3a) and second GalNAz injection (3b), scale bar: 0.1 mm.

To verify these findings and to investigate the dynamics of cupula regeneration, we injected zebrafish twice with GalNAz. Between injections the fish were returned to the aquarium tank for several days. We hypothesized that at each point in time of injection GalNAz would be incorporated into cupulin leading to two bands of labeled glycoprotein in the cupula. Figure 4 shows the result of such an experiment with a second GalNAz injection 13 days after the first one. 31 days after the second injection the fish was sacrificed and the head processed. After staining with AlexaFluor‐488 alkyne, indeed two bands were clearly visible in the cupula. The distance between the bands obviously indicates the growth of the cupula over the 13 day interval. In addition, the observation of a second band further confirms that the bands are indeed caused by GalNAz incorporation and are no artifacts.

### Complete Renewal of the Zebrafish Cupula Occurs within Approximately 8–10 Weeks

**Figure 5:**
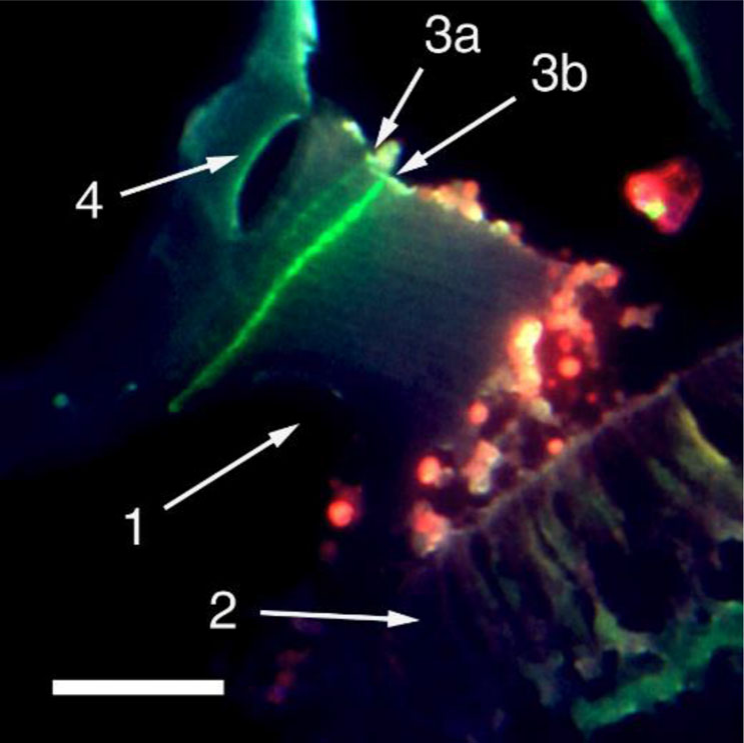
Cupula of a zebrafish after two intraperitoneal GalNAz injections with a time interval of 7 days. 50 days after the second injection, the prepared cross sections were stained with AlexaFluor‐488 alkyne. Two bands are visible, each resulting from one injection of GalNAz. The lower intensity of the upper band (resulting from the first injection) may result from a reflux of the injected substance through the hole in the abdominal wall made by the injection needle. In contrast to Figures 3 and 4, the present cupula has not separated from the ampullar roof by the shrinking process. The roof is situated at the left upper corner of the image. The time needed for the green band to reach the ampullar roof effectively illustrates the duration of complete cupula renewal. 1 = cupula, 2 = crista ampullaris, 3 = green bands from first (3a) and second GalNAz injection (3b), 4 = ampullar roof, scale bar: 0.1 mm.

To investigate the time required for complete renewal of the cupula, we carried out a long‐term experiment. A zebrafish was injected two times with GalNAz with a time delay of seven days. 50 days after the second injection, the cupula was prepared and stained. As shown in Figure 5, again two bands are visible. The upper band resulting from the first injection 57 days before sample preparation has almost reached the ampullar roof whereas the second band appears somewhat below. From this it is estimated that the complete renewal of the cupula takes about eight to ten weeks.

## Conclusion

The cupula is an acellular membrane located in each of the semicircular canals of the vestibular organ in the inner ear. It is mainly composed of the matrix glycoprotein cupulin. Since correct functioning of the cupula is essential for the detection of rotational accelerations, it has been suggested that mechanical or structural disorders are likely to be responsible for the sudden loss of vestibular function observed in humans. Employing metabolic glycoengineering, we successfully demonstrated for the first time that the cupula of adult zebrafish undergoes continual renewal. Intraperitoneal injection of GalNAz and subsequent preparation of the cupula lead to a distinct band within the cupula, visualized after fluorescence labeling with AlexaFluor‐488 alkyne. Time‐delayed double injection giving rise to two bands and long‐term experiments permits the conclusion that complete renewal of the zebrafish cupula occurs in a cycle of about eight to ten weeks.

It is remarkable that the fluorescent bands within the cupula appear narrow and clearly outlined, and that their intensity does not decrease significantly with increasing delay between GalNAz injection and sample preparation. Even after 7–8 weeks, the bands did not fade. This demonstrates clearly that the level of glycosylation of the cupula protein(s) is stable over time, which in turn indicates that the endolymph is free from glycosidases.

Since the anatomy and physiology of the semicircular canals in the vestibular organ of zebrafish are very similar to those of humans, it is likely that a comparable cupulin renewal also occurs in humans. We are aware of the fact that the constant production of cupulin implies also a mode of cupulin resorption, and we are currently investigating this aspect. Our present results provide clear evidence of a continuous cupular growth. We assume that this process prevents degeneration of this delicate sensor throughout life and could explain the spontaneous recovery as observed in patients with vestibular disorders. Disruption of cupula production might lead to defects in cupular morphology resulting in a disruption from the ampullar roof, leakage and functional deficit of the sensor, thus, providing an explanation for the occurrence of sudden loss of vestibular function. As mentioned earlier, the origin of this disease is not yet understood. These new findings on cupulin renewal and its regulation and possible disruption provide a promising starting point to direct future investigations.

## Supporting Information

The authors have cited additional references within the Supporting Information.^[^ ^47^, ^49^ ^]^

## Conflict of Interests

The authors declare no conflict of interest.

## Supporting information



Supporting Information

## Data Availability

The data that support the findings of this study are available in the supplementary material of this article.
