# Supplementary material for: Investigation Into the Dynamics of the Cupula in the Vestibular Organ of Adult Zebrafish Using Metabolic Glycoengineering
Source: Angew Chem Int Ed Engl. 2026 Jan 20;65(17):e15593. doi: 10.1002/anie.202515593 (PMC13098472; doi:10.1002/anie.202515593)
Supplement: Supplementary file 1 — Supporting Information [file ANIE-65-e15593-s001.pdf]

Supporting Information

Investigation Into the Dynamics of the Cupula in the Vestibular Organ of Adult Zebrafish Using Metabolic Glycoengineering

Hans Scherer, Andrea Jüngst, Verena F. Schöwe, Anne-Katrin Gronewald, Valentin Wittmann**

[*] Prof. Dr. H. Scherer, Department of Otolaryngology, Head and Neck Surgery and Institute of Clinical Chemistry and Pathobiochemistry, Charité-Universitäts-medizin Berlin, 13353 Berlin, Germany. E-mail: hans.scherer@charite.de

Dr. A. Jüngst (née Niederwieser), Dr. V. F. Schöwe (née Schart), Dr. A.-K. Gronewald (née Späte), Prof. Dr. V. Wittmann, Department of Chemistry and Konstanz Research School Chemical Biology (KoRS-CB), University of Konstanz, Universitätsstraße. 10 78464 Konstanz, Germany, E-mail: mail@valentin-wittmann.de

Table of Contents

Experimental Section S2

Supporting Table S4

References S4

Experimental Section

General Methods. AlexaFluor-488 alkyne was purchased from Invitrogen. GalNAz was synthesized according to Hang et al. [1] and used as a 250 mM stock solution in phosphate buffered saline (PBS). The animal experiments were approved by the authorities of Berlin (Landesamt für Gesundheit und Soziales, LAGeSo) and the Charité-Universitätsmedizin (permission number G 0148/13). Zebrafish were obtained from the Bundesinstitut für Risikobewertung, Alt Marienfelde 19-21, 12277 Berlin and held by the author H.S. at the Institute of Biochemistry, Charité-Universitätsmedizin authorized by LAGeSo Berlin under the number ZH 177. Fish included in this investigation had weights between 0.4 g and 0.6 g.

Fish Experiments. Wildtype zebrafish to be treated were separated in an additional tank for at least 2 days before carbohydrate injection and kept without feeding for this time. Fish to be injected were anesthetized in an aqueous tricaine (MS 222) solution (100 mg L^{-1}). The temperature of this solution was slowly lowered until the fish showed no reaction to touch. Then, the fish were transferred to an electronic micro-weighing device for scaling and, subsequently, to an operating place (wet soft sponge with an incision in the middle).[2] The incision was opened and the fish placed with the belly up between the walls of the incised sponge. Intraperitoneal injection of GalNAz (250 mM in PBS) (approx. 5 µL per gram fish) was carried out with a 10 µL syringe (WPI nanofil 01C) and a 34-gauge beveled needle (WPI NF34BV) from the location between the abdominal fins in a dorso-cranial direction. A surgical microscope (Leica/Wild M650) was used for this procedure. Thereafter, the fish were returned to the tank and kept with their mouths against the flow of oxygenated water until spontaneous movements could be noticed. Experimental parameters for each individual experiment are summarized in Table S1.

Preparation of Cross Sections. For histology, the fish was anesthetized in the above-described tricaine solution. After narcosis was reached, the fish was transferred to a concentrated tricaine solution until movements of the gills stopped showing that the fish was dead. After decapitation, the head was kept in 4 % formaldehyde for 24 h and in EDTA solution for 7 days. After paraffin embedding, cross sections were made beginning from the lateral side of the head (sagittal plane). Native sections were deparaffinized with air of a temperature of 60 °C for 30 min and

with xylene for 3 min. The slices were cleaned two times with ethanol (100 % and 70 %, respectively).

Staining and Fluorescence Microscopy. The slices were kept in PBS for 30 min. Click reactions were carried out using “Click-iT® Cell Reaction Buffer Kit, C10269” from Invitrogen. For the preparation of 1 mL of click reaction mix, 897 µL 1x Click-it Reaction buffer, 1 µL $CuSO_4$ (100 mM), 2 µL AlexaFluor-488 alkyne (1 mM in DMSO) (A10267, Invitrogen) and 100 µL reaction buffer additive were mixed and the cross sections were incubated with the reaction mixture for 30 min at room temperature. The slices were washed twice for 30 min with PBS, cleaned with ethanol, and kept in darkness. Visualization was performed with a fluorescence microscope (Zeiss Axio Z1 and MetaSystems ISIS) using the DFR filter combination. AlexaFluor-488 fluorescence was detected with the FITC filter set. The fish anatomy was visualized with the Dapi and Rhod filter sets. It should be mentioned that the autofluorescence of adult zebrafish is very high in comparison to the commonly used embryos. Thus, the pictures are very colorful.

Supporting Table

Table S1. Body weights of fish and details on sugar injection and fluorescence labeling for experiments shown in Figures 3–5.

Fig.	fish code	body weight	amount injected	time delay between first (and second) injection and sacrifice	click reaction
3A	Z 4	0.4 g	2 μL	4 d	+
3B	Z 20a	0.4 g	2 μL	4 d	+
3C	Z 4	0.4 g	2 μL	4 d	–
3D	Z 5	0.6 g	3 μL[a]	4 d	+
4	Z II 3	0.4 g	2 x 3 μL	44 d, 31 d	+
5	LV 1-4	0.6 g	2 x 3 μL	57 d, 50 d	+

[a] In this control experiment, PBS only was injected.

References

[1] H. C. Hang, C. Yu, D. L. Kato, C. R. Bertozzi, "A metabolic labeling approach toward proteomic analysis of mucin-type O-linked glycosylation" *Proc. Natl. Acad. Sci. U. S. A.* **2003**, *100*, 14846-14851.

[2] M. D. Kinkel, S. C. Eames, L. H. Philipson, V. E. Prince, "Intraperitoneal injection into adult zebrafish" *J. Visualized Exp.* **2010**.
